# Point-of-Care Ultrasound Diagnosis of Pneumoperitoneum in the Emergency Department

**DOI:** 10.7759/cureus.8503

**Published:** 2020-06-08

**Authors:** Melissa Bacci, Roli Kushwaha, Gabriel Cabrera, Eric J Kalivoda

**Affiliations:** 1 Emergency Medicine, Hospital Corporation of America West Florida Graduate Medical Education Consortium/Brandon Regional Hospital, University of South Florida Morsani College of Medicine, Brandon, USA

**Keywords:** point-of-care ultrasound, ultrasonography, pneumoperitoneum, intraperitoneal free air, gastrointestinal perforation, perforated viscus, acute abdomen, emergency department

## Abstract

Prompt and accurate diagnostic evaluation of the nontraumatic acute abdomen in the emergency department (ED) is crucial to lessen mortality burden. In patients with perforated viscus and pneumoperitoneum, point-of-care ultrasound (POCUS) can assist the emergency physician (EP) in the rapid bedside diagnosis. This report describes a case in which EP-performed POCUS led to the early detection and timely management of an atypical presentation of pneumoperitoneum.

## Introduction

The critical management of a patient presenting with acute abdominal pain in the emergency department (ED) necessitates rapid diagnostic evaluation, especially given the substantial morbidity- and mortality-associated sequela of these clinical conditions [1,2]. Gastrointestinal perforation with resultant pneumoperitoneum (intraperitoneal free air secondary to perforated viscus) is an important etiology of acute abdomen cases [3]. Point-of-care ultrasound (POCUS) has a significant and effective role in the prompt bedside diagnosis of many causes of acute abdomen, specifically perforated viscus and pneumoperitoneum [4,5]. The sonographic detection of pneumoperitoneum was initially described as the ‘Enhanced Peritoneal Stripe Sign (EPSS)’, which involves identification of focal hyperechogenic thickening of the peritoneum with associated posterior dirty shadowing and/or horizontal reverberation artifacts [6-8]. The POCUS findings of pneumoperitoneum have been reported with notably high accuracy; thus, it is an ideal imaging modality for the emergency physician (EP) requiring a timely diagnosis [9,10]. Several reports have previously highlighted the value of EP-performed POCUS to diagnose pneumoperitoneum in patients with hemodynamic instability and/or clinical presentations highly suggestive of perforated viscus [11-14]. This case report describes the utility of EP-performed POCUS in the early recognition of pneumoperitoneum for the initial evaluation of a patient with an atypical presentation for a life-threatening gastrointestinal perforation.

## Case presentation

A 52-year-old male with no reported past medical or surgical history presented to the ED with three days of insidious, mild-severity generalized vague abdominal pain with associated nausea, nonbloody nonbilious vomiting, and decreased appetite. He denied fevers, chills, diarrhea, constipation, dark or bloody stools, flank pain, back pain, testicular pain or swelling, genital lesions or discharge, urinary complaints, or any other associated symptoms. Upon arrival, the patient was afebrile, with a blood pressure of 144/102 mmHg, heart rate 114 beats per minute, respiratory rate 16 breaths per minute, and oxygen saturation of 95% on room air. On physical examination, he was in mild distress and had mild diffuse abdominal tenderness to palpation without rebound, guarding, or peritoneal signs. Of note, examination was unremarkable for scleral icterus, flank or costovertebral angle tenderness, or clinical signs of significant dehydration. 

**Figure 1:**
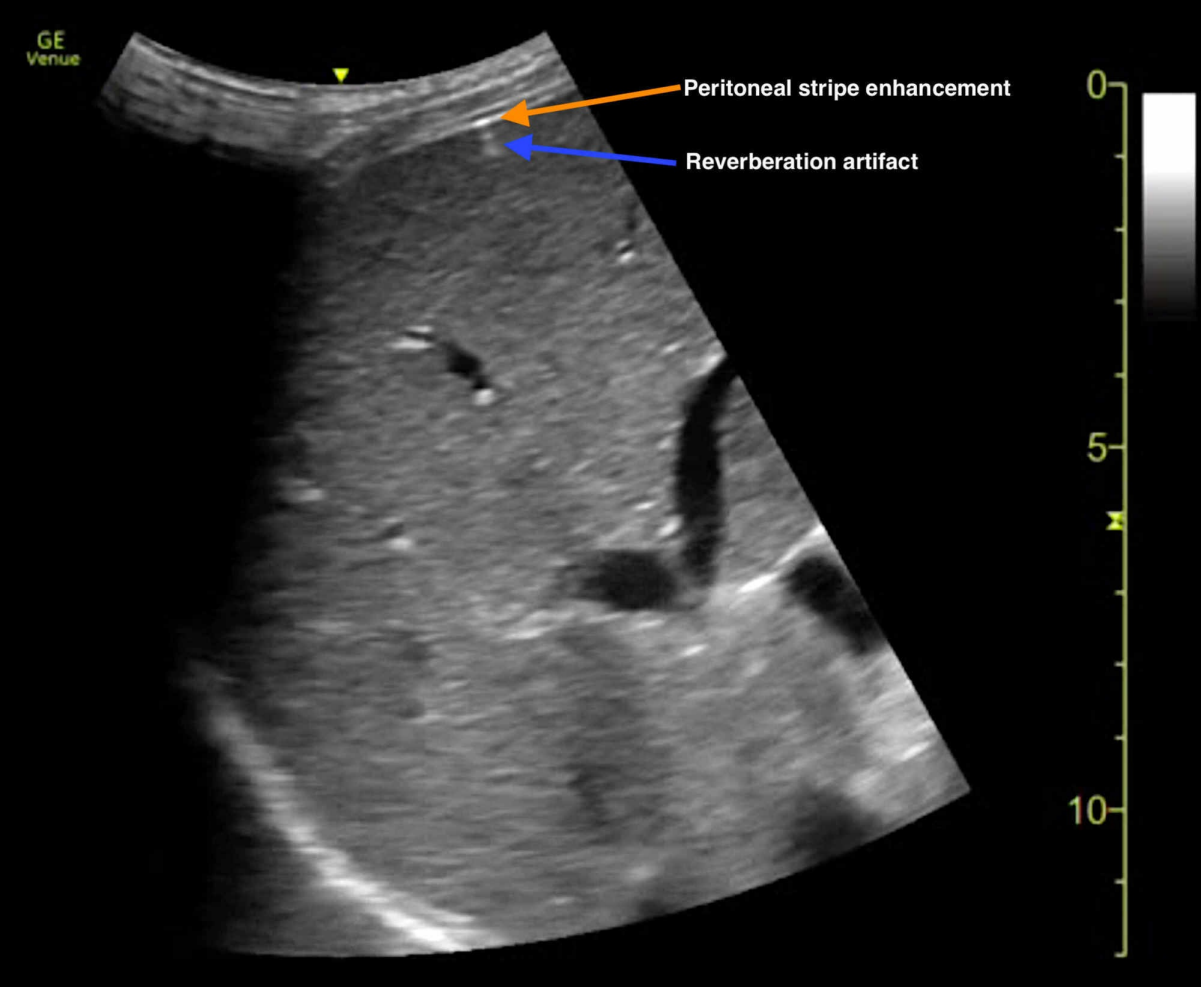
Point-of-care ultrasound diagnosis of pneumoperitoneum. Gas bubble abutting the liver and abdominal wall is indicated by peritoneal stripe enhancement (orange arrow) with corresponding reverberation artifact (blue arrow).

His clinical presentation was relatively nonspecific. However, there was concern for an acute abdominal pathology due to his clinical history and examination. CT imaging of the abdomen/pelvis, laboratory studies, pain medications, intravenous (IV) fluids, blood cultures and broad-spectrum IV antibiotics were ordered. POCUS was subsequently performed by an ultrasound fellowship-trained EP attending and emergency medicine resident physicians. POCUS assessment of the right hypochondrium with a curvilinear transducer in the sagittal plane demonstrated sonographic findings consistent with pneumoperitoneum; focal enhancement of the peritoneal stripe between the liver and abdominal wall and associated reverberation artifacts were appreciated (Figure 1) (Video 1).

**Video 1:** Point-of-care ultrasound diagnosis of pneumoperitoneum.

**Figure 2:**
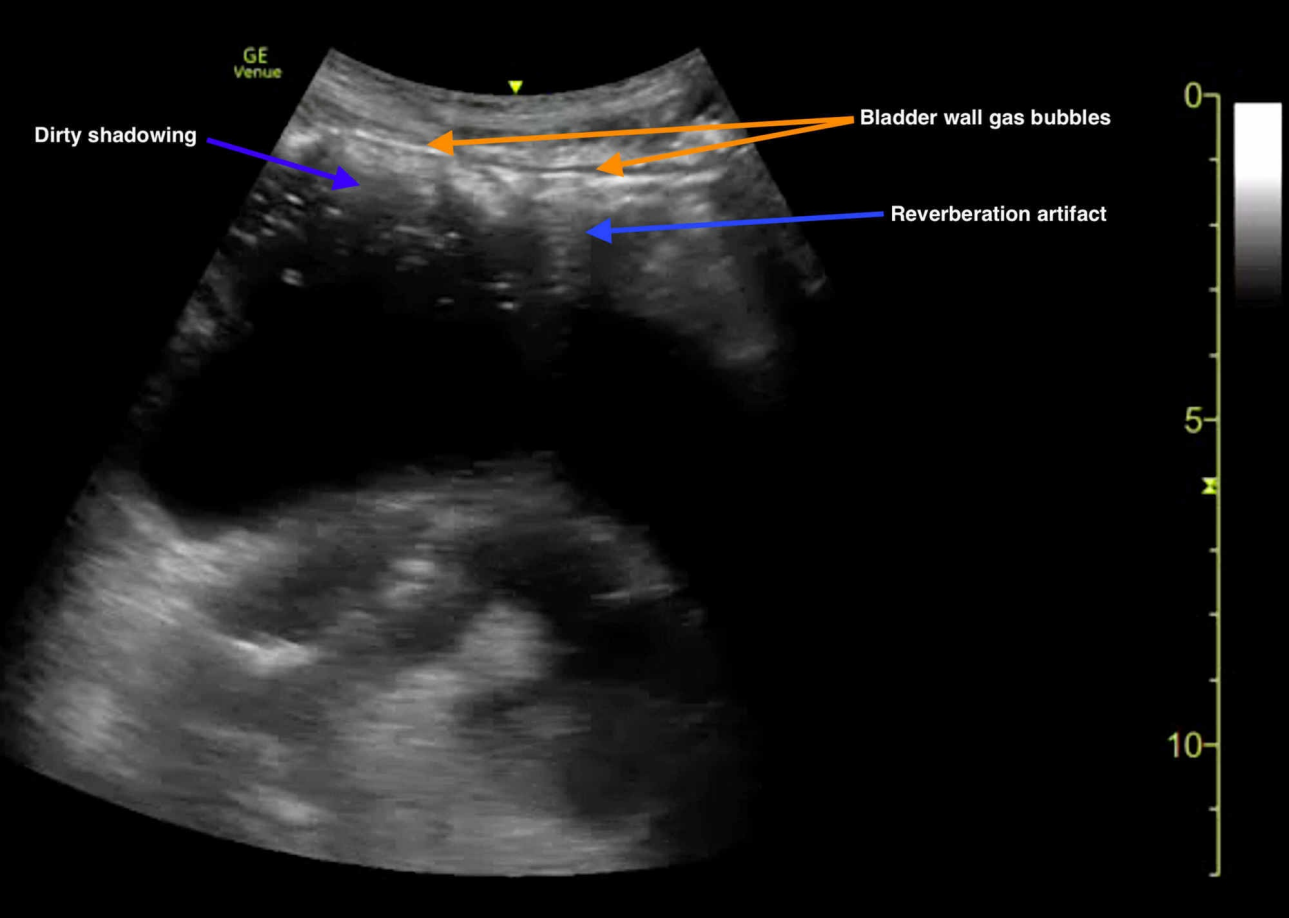
Point-of-care ultrasound demonstrating an air-fluid level of the bladder. Gas bubble of anterior bladder wall is indicated by multiple hyperechoic foci (orange arrows) with corresponding reverberation artifact and dirty shadowing (blue arrows).

POCUS assessment of the bladder demonstrated sonographic evidence of air accumulation, with multiple hyperechoic foci in the anterior bladder wall and associated reverberation artifacts and/or dirty posterior shadowing (Figure 2) (Video 2). Of note, there were no contributing pathologies identified on POCUS evaluation of the gallbladder, kidneys, hepato-renal space, spleno-renal space, or abdominal aorta. 

**Video 2:** Point-of-care ultrasound demonstrating an air-fluid level of the urinary bladder.

**Figure 3:**
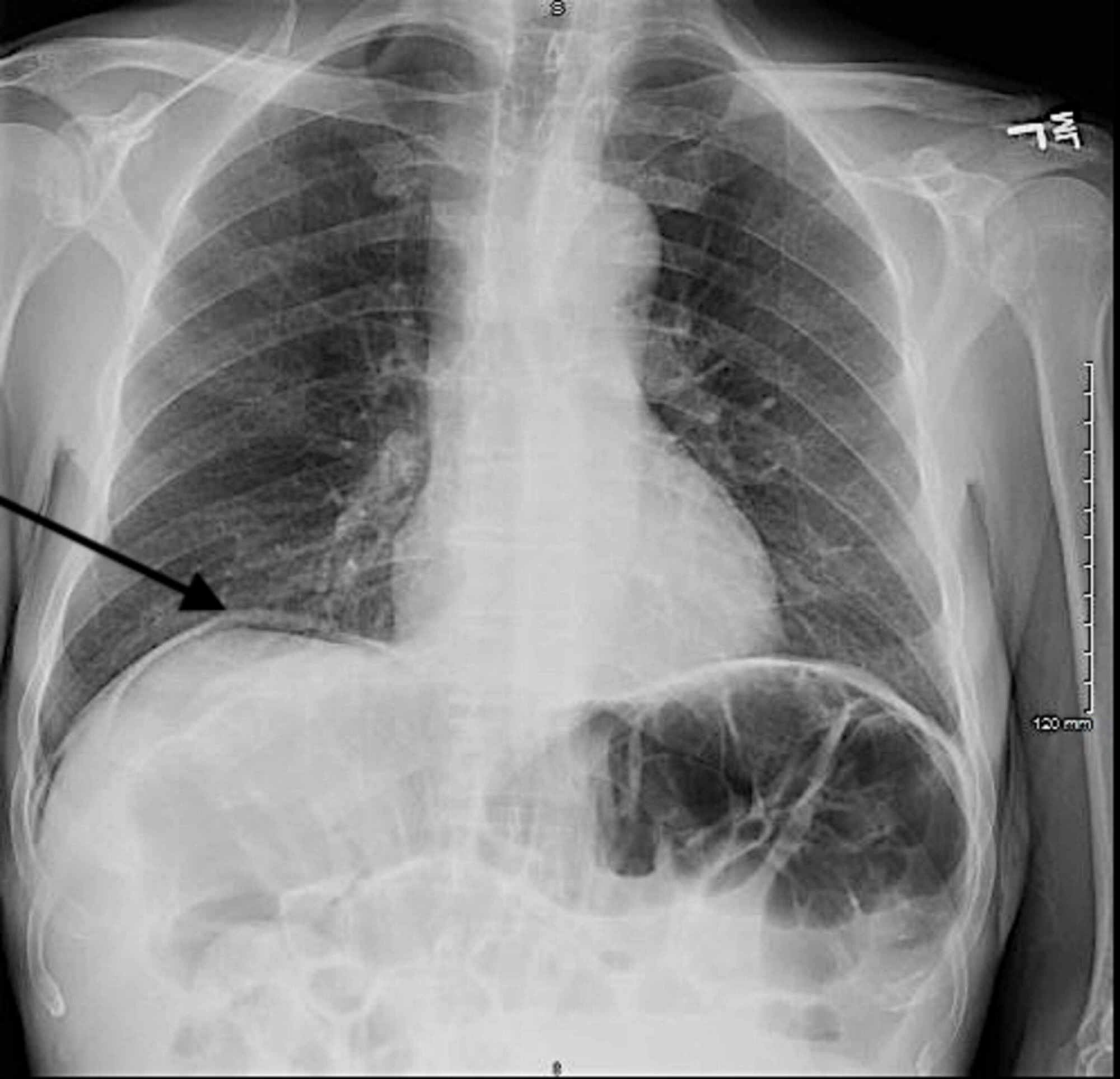
Portable chest radiography demonstrating free air under the diaphragm on the right (black arrow) consistent with bowel perforation.

Portable chest radiography was then performed due to the abnormal POCUS findings, which confirmed the presence of intra-abdominal free air on the right consistent with bowel perforation (Figure 3). General surgery was immediately consulted, and surgical preparations were made for emergent exploratory laparoscopy. Laboratory analysis was notable for white blood cell count of 25.0 x 10^3^/μL and a venous lactate of 2.0 mmol/L. 

**Figure 4:**
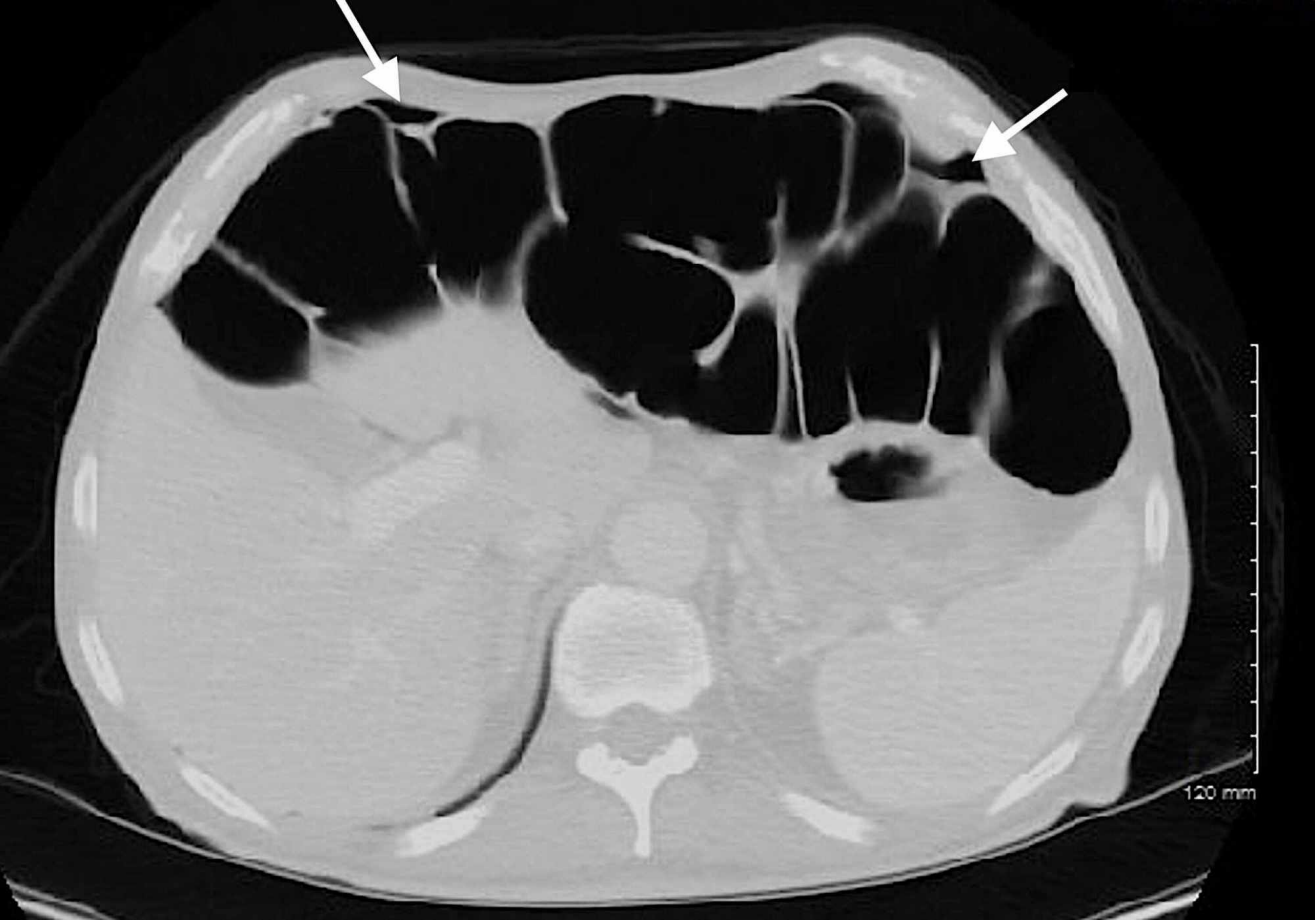
CT of the abdomen/pelvis demonstrating intraperitoneal free air (white arrows).

**Figure 5:**
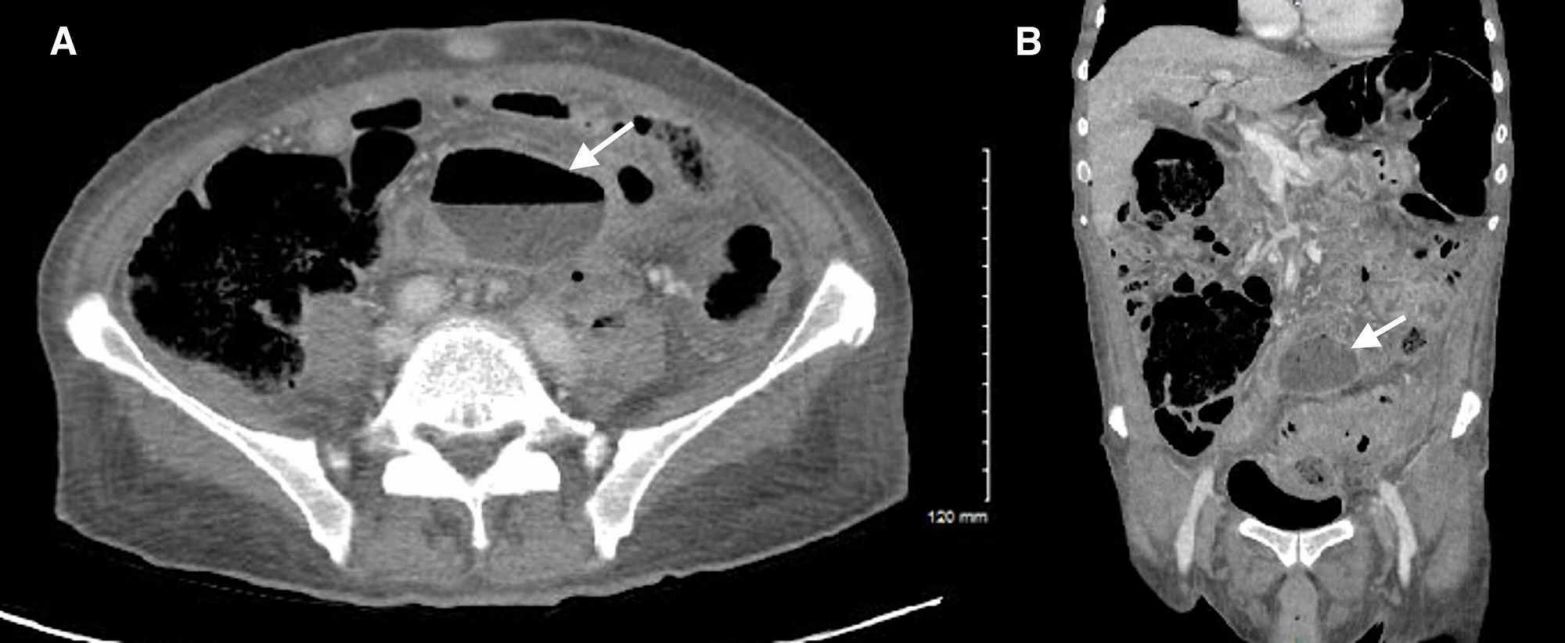
CT of the abdomen/pelvis demonstrating an intra-abdominal abscess collection #1 (white arrows) secondary to sigmoid colon perforation. Axial (A) and coronal (B) planes.

**Figure 6:**
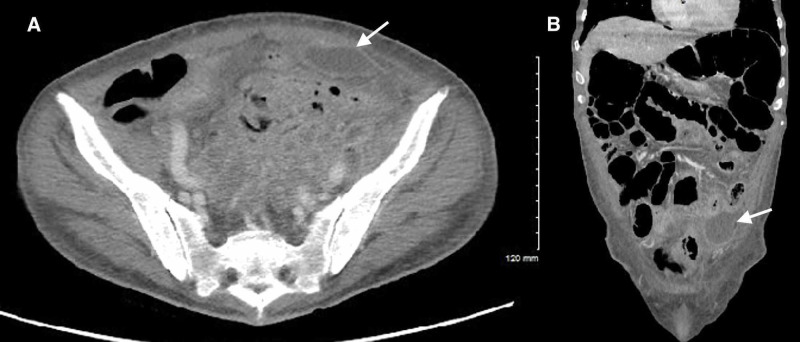
CT of the abdomen/pelvis demonstrating an intra-abdominal abscess collection #2 (white arrows) secondary to sigmoid colon perforation. Axial (A) and coronal (B) planes.

**Figure 7:**
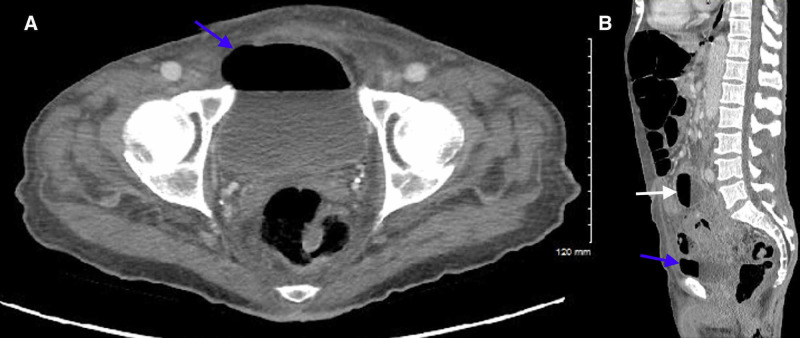
CT of the abdomen/pelvis demonstrating air in the urinary bladder (blue arrows) and intra-abdominal abscess collection #1 (white arrow). Axial (A) and sagittal (B) planes.

CT of the abdomen/pelvis was obtained en route to the operating room, which ultimately demonstrated intraperitoneal free air, an air-fluid level in the bladder suggesting prior instrumentation or fistula, and sigmoid diverticulitis complicated by multiple abscess collections secondary to sigmoid colon perforation (Figures 4-7). The postoperative surgical report confirmed the diagnoses of perforated sigmoid colon, large pelvic abscesses, and a colovesicular fistula for which a partial colectomy with end colostomy and takedown of colovesicular fistula was performed. The patient was ultimately discharged home in stable condition with instructions for outpatient surgery and urology follow-up. 

## Discussion

Prompt diagnosis can expedite the emergent management of pneumoperitoneum secondary to perforated viscus. EPs can harness the multiple advantages of POCUS in scenarios of acute abdominal pain, chiefly for the rapid primary evaluation and necessary re-assessments depending on the clinical presentation. The conventional practice of initially obtaining plain radiographs for pneumoperitoneum can be of limited diagnostic value in the ED, while multiple studies have highlighted the superior diagnostic sensitivity of ultrasonography compared to plain radiography [15-17]. The diagnostic performance of ultrasonography versus plain radiography for pneumoperitoneum has been reported with sensitivity (93% versus 79%), accuracy (90% versus 77%), specificity (64% versus 64%), and positive predictive value (97% versus 96%) [17]. Our patient case demonstrated early bedside sonographic evidence of pneumoperitoneum before any other imaging modalities were obtained, which was significant in that emergent surgical intervention proceeded without delay. CT imaging remains an essential component of the ED diagnostic algorithm in evaluation of perforated viscus, predominantly for its role in downstream surgical planning [18]. There is certainly an emerging role for EP-performed POCUS in the identification of pneumoperitoneum in the ED.

EP training in advanced POCUS for gastrointestinal pathologies is of critical importance [19]. In our opinion, it is imperative that emergency medicine providers are highly skilled in applying the described POCUS techniques for the ED management of suspected cases of pneumoperitoneum. Furthermore, routinely integrating POCUS evaluation of pneumoperitoneum into abdominal-pelvic trauma scenarios has been previously suggested; pneumoperitoneum assessment could be incorporated into the secondary survey as an adjunct to the focused assessment with sonography for trauma (FAST) examination [20]. This case report illustrates the significance of utilizing POCUS in guiding the collaboration of EPs and surgical consultants in the management of perforated viscus.

## Conclusions

Perforated viscus with associated pneumoperitoneum is a life-threatening etiology of acute abdominal pain in the ED. EP-performed POCUS provides an invaluable tool to rapidly diagnose pneumoperitoneum and expedite surgical consultation. Further studies are required to determine the most effective role(s) and timing of POCUS in ED evaluation of pneumoperitoneum in both atraumatic and traumatic clinical situations.
